# Leg Venous Properties in Children With Myelomeningocele

**DOI:** 10.3389/fped.2020.00531

**Published:** 2020-09-02

**Authors:** Małgorzata Domagalska–Szopa, Andrzej Szopa, Maximilian Puchner, Lutz Schreiber, Andrzej Siwiec, Magdalena Hagner-Derengowska, Damian Ziaja

**Affiliations:** ^1^Department of Medical Rehabilitation, School of Health Sciences in Katowice, Medical University of Silesia, Katowice, Poland; ^2^Department of Physiotherapy, School of Health Sciences in Katowice, Medical University of Silesia, Katowice, Poland; ^3^Department of Neurosurgery, Klinikum Vest, Academic Teaching Hospital, Ruhr University Bochum, Bochum, Germany; ^4^Pediatrics Center John Paul II in Sosnowiec, Sosnowiec, Poland; ^5^Faculty of Earth Sciences and Spatial Management, University Nicolaus Copernicus, Torun, Poland

**Keywords:** spina bifida, spinal cord malformations, spine, duplex scanning, venous reflux

## Abstract

**Introduction:** The vascular properties of individuals with myelomeningocele (MMC) are an underestimated problem, as evidenced by the lack of relevant research. Therefore, this study was conducted to assess the venous properties of the leg in children with MMC. This study compared the duration of retrograde flow (RF) of the distal and proximal sites of the great saphenous vein (GSV) in children with MMC and typically developing (TD) children. Additionally, the impact of MMC clinical features, such as the anatomical level of the spinal cord defect, muscle strength of the lower limbs, and level of gross motor functional abilities on the of GSV sufficiency were assessed.

**Methods:** Thirty ambulant children between 7 and 12 years with MMC and an age- and sex-matched sample of thirty children with typical development (TD) were included in the study. All participants underwent a complete physical examination that included gross motor assessment, manual muscle testing, and duplex ultrasound examination of the GSV reflux. The duration of retrograde flow (RT) in the GSV was evaluated at four sites: P1: proximal thigh; P2: medial thigh; P3: upper leg; and P4: lower leg. The measurements were performed in two body positions: horizontal position (HP) and vertical position (VP).

**Results:** Children with MMC showed increased duration of RT of both the proximal and peripheral sites of GSV, as compared with the TD peers. The prevalence of GSV reflux in peripheral segments was significantly higher than in the proximal segments. The severity of MMC (expressed by higher level of the spinal cord defect), deficit of thigh and leg muscle strength, and lower functional independence negatively influenced the GSV sufficiency in patients with MMC. Gravity directly influenced GSV reflux occurrence and reflux hemodynamic parameters in MMC.

**Conclusion:** These findings may help better understand aspects concerning the risk of developing venous insufficiency in children with MMC and determine better screening, prevention, and treatment algorithms for venous insufficiency in patients with SB.

## Introduction

A neural tube defect is defined as any condition that involves incomplete development of the brain, spinal cord, and/or their protective coverings. Spina bifida (SB) is a congenital disorder classified as a neural tube defect and is caused by incomplete closure of the fetal spinal column during the first month of pregnancy (1). Infants born with SB can have only a spina bifida occulta or closed spinal dysraphisms without visible neurological lesions. However, sometimes they have an open lesion on their spine included damage to the nerves and spinal cord, which can result in varying degrees of lower limb paralysis and numerous accompanying symptoms depending on the level of the lesion and forms of myelodysplasia (1).

The extent of the impairment is directly related to the size and location of the neural tube defect and the emerging spinal nerves from the affected area of the spinal cord. The higher the level of the lesion, the greater the degree of muscular impairment (1, 2).

Spinal cord malformation occurs in varying degrees of severity. There are three types of SB: occulta, spina bifida cystica (meningocele), and myelomeningocele (MMC). Occulta is the mildest and most common form. In this defect, the vertebral arches are unfused; however, there is no herniation or displacement of neural tissues, and only skin changes over the defect are observed. Neurological signs may be present. This form of SB does not usually cause disability or show symptoms, and most commonly involves the L5 and S1. In meningocele, the vertebral arches are unfused; the meninges are pushed out from the spinal opening and may be covered by a layer of skin. Some patients show few or no symptoms, while in others, the malformation causes partial paralysis with urinary and bowel problems (2). Myelomeningocele—the most severe form of SB—occurs when the spinal cord is pushed through the opening in the spine resulting in partial or complete paralysis of the bodily areas below the spinal opening (2). If the nerve roots are damaged or the spinal cord is dysplastic, the patient will show classic lower motor neuron presentation with a flaccid type of motor paralysis and lack of sensation. If part of the spinal cord below the MMC is intact and has innervation, the result is a spastic type of motor paralysis (2). MMC at higher levels results in more severe neurological and orthopedic outcomes and is associated with more severe brain lesions and motor and cognitive impairments than those at lower levels (2).

The most common area of MMC is the lumbosacral region. The primary clinical features manifested in a child with a lumbosacral MMC are flaccid paralysis (muscle weakness and wasting), decreased or absent tendon reflex, decreased or absent exteroceptive and/or proprioceptive sensation, and paralytic and congenital deformities. It is often accompanied by numerous secondary symptoms such as rectal and bladder incontinence, soft tissues contractures, bone deformity, and osteoporosis (3, 4).

A spinal cord injury is known to result in paralysis and cause concomitant muscle atrophy; consequently, blood flow below the level of the lesion will decrease. Some studies observed decreased blood flow in the femoral artery of spinal cord-injured vs. able-bodied individuals (5). Other studies have shown that deteriorating vascular properties (small diameter, low flow, high shear stress) are also present in adults with SB (5–7). Children with MMC have a higher risk of severe vasomotor changes and peripheral blood circulation disorders. They are also prone to secondary complications because of poor circulation, for example, decubitus and poor wound healing, pressure ulceration of the skin, and difficult-to-treat deep wounds or infections (5, 7). To our best knowledge, the vascular properties of individuals with MMC have never been studied. Further, blood circulation disorders in children with MMC are an underestimated problem, as evidenced by the lack of relevant research. Therefore, this study was conducted to assess the venous properties of the leg in children with MMC. We hypothesized that venous insufficiency occurs in children with MMC.

The primary objective of this study was to examine the venous properties of the leg in children with MMC with duplex scanning. We compared the duration of retrograde flow (RF) of the distal and proximal sites of the great saphenous vein (GSV) in children with MMC and typically developing (TD) children. The secondary objectives included evaluation of the relative impact of MMC clinical features, such as the anatomical level of the spinal cord defect, muscle strength of the lower limbs, and level of gross motor functional abilities on the of GSV sufficiency.

## Participants

Thirty ambulant children (age mean was 8.8 years, age range, 7–12 years) with MMC who were patients of local pediatric rehabilitation centers were included in the study. All participants met the following criteria: (1) a diagnosis of MMC in the lumbosacral region; (2) age range, 7–12 years; (3) ability to follow verbal directions; (4) ability to maintain sitting and standing position with/without support; and (5) the possibility of accessing the venous examination areas being studied (groin, popliteal space) by an ultrasound handheld device. The exclusion criteria were as follows: (1) presence of other neurological lesions or malformations or chronic diseases not related to MMC; (2) allergy to gel used for Doppler ultrasound examination; (3) hip flexion contracture >30 degrees in any one lower extremity; and (4) impossibility to access the aforementioned examination areas by an ultrasound handheld device. An age- and sex-matched sample of 30 children with typical development (TD) and no known history of neurological or orthopedic diseases were included as controls in the study. No participant was treated for venous disease.

## Methods

The study was approved by The Ethical Committee of the Medical University of Silesia. The children were subjected to examinations after obtaining parental informed written consent. All work was performed in accordance with the Code of Ethics of the World Medical Association (Declaration of Helsinki). All patients underwent a complete physical examination that included gross motor assessment, manual muscle testing, duplex ultrasound examination of the GSV reflux.

### Functional Motor Assessment

Motor function was assessed using the Gross Motor Function Measure (GMFM-88) score sheet to quantify motor skills. The GMFM−88 scale span the spectrum of functional activity from lying and rolling up (A; 17 items) to sitting (B; 20 items); crawling and kneeling (C; 14 items); standing (D; 13 items); walking, running, and jumping (E; 24 items) skills. Each of five items is scored in four-point system, from 0 (cannot perform) to 3 (normal performance). GMFM-88 item scores were summed and expressed as percentages total GMFM-88 score (8).

### Manual Muscle Testing

Manual Muscle Testing (MMT) is a method to check the muscular strength. Each child underwent MMT of the following five muscle groups in each limb: (1) Hip flexors, (2) Knee extensors, (3) Ankle dorsiflexors, (4) Long toe extensors, and (5) Ankle plantar flexors [according to the American Spinal Injury Association [ASIA] Motor Impairment Scale] (Table 1) (9). The testing positions and standardization procedures for all measurements were in accordance with the International Myelodysplasia Study Group (IMSG) recommendation for MMT in people with myelodysplasia (Table 2) (10).

**Table 1:** Key myotomes and dermatomes for lower extremity neurologic testing, according to the American Spinal Injury Association (ASIA) Impairment Scale.

**Root**	**Functional group**	**Myotome**
L2	Hip flexors	Iliopsoas
L3	Knee extensors	Quadriceps
L4	Ankle dorsiflexors	Tibialis Anterior
L5	Long toe extensors	Extensor halluces longus
S1	Ankle plantar flexors	Gastrocnemius & Soleus

**Table 2:** Manual Muscle Testing (MMT).

**Score**	**Muscular strength**	**Muscle activity**
0	Zero	No evidence of contractility; total paralysis
1	Trace	Evidence of slight contractility (no joint motion); palpable or visiblecontraction
2	Poor	Active movement; full range of motion with gravity eliminated
3	Fair	Active movement; full range of motion against gravity
4	Good	Active movement; complete range of motion against gravity and moderate resistance in a muscle-specific position
5	Normal	Complete range of motion against gravity with full resistance in a functional muscle position expected from an otherwise unimpaired person

### Doppler Ultrasound Examination

After completing the GMFM and MMT assessment, subjects were positioned supine on an examination table for duplex scanning to determine venous reflux.

Ultrasound examination of GSV was performed by the same experienced examiner (vascular surgeon) in a single experimental room between 1,100 and 1,400 h, and the temperature was maintained between 22 and 24°C.

The ultrasound examination of GSV was performed according to the Guideline for the Performance of Peripheral Venous Ultrasound Examinations (11, 12).

After a provocation maneuver, the duration of retrograde flow (RT) in the GSV was evaluated at the following four different points:

P1: proximal thigh (near the saphenofemoral junction; SFJ)
P2: medial thigh (20 cm above the knee fold)
P3: upper leg (4 cm under the knee fold)
P4: lower leg (in the medial malleolus region).

Duplex scanning for GSV were performed separately on both lower limbs. Prior the examination GSV sites were marked. The measurements were performed in two body positions: (1) horizontal position (HP), i.e., lying position on an examination table with adjustable height, and (2) vertical position (VP) on the platform with handrails for patients' stability. Patients were initially placed in HP in the reverse Trendelenburg position (i.e., supine position with elevated head and torso on wedge). This position is recommended as it facilitates venous filling in the lower extremities and causes dilation of veins (11). The lower limb position in external rotation of the hip and slight flexion of the knee helps to decrease muscle tension and is suitable both for exposing the deep veins in the medial thigh and posterior knee as well as for the compression maneuver (11, 12).

Rapid-inflation pneumatic cuffs with a maximum pressure of 80 mmHg were used to augment flow. The same pressure (80 mmHg) was used with subjects in both the horizontal and vertical positions (13). Time to inflation was 0.3 s, inflation was maintained for 1 s, and deflation was achieved in <1 s (after Labropoulos 13). For P1 and P2 measurements, the cuff was placed on the lower thigh, while for P3 and P4, it was placed on the lower calf. After completion of all measurements in HP, patients were moved to VP.

The VP refers to a standing position for children in any of the following scenarios: able to maintain this position without support, with support on handrails, or with the support of another person when the position could not be maintained independently. In both cases patients under-loaded the examined leg, to ensure maximum venous distention on examined one.

After a 5-min rest, the same measurements were repeated. Detection and measurement of the duration of RT was performed with duplex ultrasound with pulsed wave Doppler (SonoScape Expert 8 ultrasound machine with color flow imaging). A linear probe with Doppler effect was used (Linear probe 5–7, 5 MHz L743).

### Data Analysis

Patients were classified as high lumbar (A); low lumbar (B); or sacral (C) according to their neurological lesion level. For all participants, the MMT score was recorded on a scale between 0 and 5, with the possibility of a half point, e.g., a score of 3.5. The motor examination consisted of grading seven specific muscle groups in the lower extremities, representing major lumbar myotomes (Table 3). Motor strength was graded using a universal six-point scale (graded as 0–5). Bilateral motor strength was recorded for each muscle group. The RT (on second) of GSV (at four different points) of 60 lower limbs each of 30 children with MMC and 30 TD children were measured. No patient had any missing data.

**Table 3:** Demographic characteristics of children with myelomeningocele (MMC group) and typical developing children (TD group).

**Characteristics**	**MMC (*n* = 30)**	**TD (*n* = 30)**	**Statistical tests *P*-values**
Age (years), mean (SD)	8.8 (3.5)	8.1 (3.7)	*U* = 395; *P* = 0.42
Gender, boys n (%)	19 (63)	14 (64)	χ^2^ = 0.0; *P* = 1.0
Weight (kg), mean (SD)	32.4 (10.0)	33.8 (11.1)	*U* = 428; *P* = 0.76
Height (cm), mean (SD)	125.0 (15.2)	133.0 (18.3)	*U* = 349; *P* = 0.14
BMI (kg/m^2^), mean (SD)	20.3 (3.2)	17.4 (3.2)	*U* = 229; *P* = 0.001
BMI percentile, mean (SD)	81.6 (22.5)	54.6 (28.3)	*U* = 191; *P* = 0.001
Functional characteristics GMFCS levels			
I, n (%)	11 (37)		
II, n (%)	13 (43)		
III, n (%)	6 (20)		
GMFM score %, median (range)	53.6 (28.3–72.2)		
Neurological characteristics lesion level			
(A) High lumbar, n (%)	12 (40)		
(B) Low lumbar, n (%)	10 (33)		
(C) Sacral, n (%)	8 (27)		
Secondary complications			
Bladder/bowel dysfunction yes n (%)	10 (20)		
Lower limbs edema, yes n (%)	15 (50)		
Pressure ulcers and poor wound healing, yes n (%)	13 (43)		

*U = Mann–Whitney test; χ^2^ = chi-square test*.

*Means (SD) of the outcome (95% CI), comparison between the groups (statistical tests and P-values), and functional and neurological characteristics of the children with MMC*.

### Statistical Analysis

The software package Statistica 12.0 PL was used to carry out statistical analyses. The normality of the distribution of analyzed parameters was assessed using skewness and kurtosis and the Shapiro–Wilk test. Descriptive statistics were calculated for the clinical characteristics of both the MMC and TD groups. Differences in mean duration of retrograde flow (RT) in the GSV in HP and VP were assessed between the MMC groups and TD using U = Mann–Whitney *U*-test for independent samples. The Spearman rank correlation test was used to examine the relationship between RT of GSV in both position and lower limbs muscle strength scores, and gross motor function scores (GMFM). The correlations were performed only for children with MMC. Coefficients with a *P* < 0.05 were considered significant. The correlations were interpreted according to the guidelines adopted from Altman: Rs <0.2, poor; 0.21–0.4, fair; 0.41–0.6, moderate; 0.61–0.8, good; and 0.81–1, very good (14).

## Results

Table 3 shows the demographic information for both groups (age, sex, weight, height, BMI, and BMI percentile). Tests for normality were conducted, and all data showed non-normal distribution. Patients in both groups were comparable for age, sex, weight, and height (*P* > 0.2). BMI and BMI percentile index were significantly different in the MMC compared to the TD group (Table 3). Among MMC patients, only 7 (23%) had normal BMI, 13 patients (43%) were overweight, and 10 patients (33%) were obese.

The functional characteristics of the MMC group are also shown in Table 3. In the MMC group, the children predominantly showed levels I and II in GMFCS (37 and 43%, respectively); the remaining 20% were evaluated as level III GMFCS.

The MMT scores are shown in Table 4. Children with MMC showed about two-times poorer muscle strength of the lower limbs of all examined muscle groups than TD children.

**Table 4:** Muscle strength of lower limbs in children with myelomeningocele (MMC group) and typical developing children (TD group).

**Muscle group**	**MMC (*****n*** **=** **60)**			**TD (*****n*** **=** **60)**			**Statistical test**
	**Mean (SD)**	**Median**	**Range**	**Mean (SD)**	**Median**	**Range**	***P*-values**
HF	2.75 (1.07)	3.00	0.0–4.5	4.63 (0.41)	4.75	4.0–5.0	*U* = 3.47; *P* < 0.001
KE	2.46 (1.34)	3.00	0.0–4.5	4.64 (0.37)	4.50	4.0–5.0	*U* = 3.52; *P* < 0.001
ADF	1.91 (1.52)	1.50	0.0–4.5	4.95 (0.15)	5.00	4.5–5.0	*U* = 3.59; *P* < 0.001
LTE	1.55 (1.11)	2.00	0.0–3.5	4.72 (0.31)	4.75	4.0–5.0	*U* = 3.60; *P* < 0.001
APF	0.82 (0.91)	0.50	0.0–3.0	4.74 (0.31)	5.00	4.0–5.0	*U* = 3.60; *P* < 0.001

*HF, Hip flexors; KE, knee extensors; ADF, ankle dorsiflexors; LTE, long toe extensors; APF, ankle plantar flexors; U = Mann–Whitney test for independent samples*.

*Means (SD), median, and range of the outcome (95% CI) and comparison between the groups (statistical tests and P-values)*.

The results of the duration of RT assessments in the GSV of two proximal (P1 and P2) and two distal segments (P3 and P4), i.e., four segments for 60 lower limbs (240 cases) in two positions (HP and VP; 480 cases) in each group of participants (MMC and TD) are presented in Table 5. In all comparisons, children with MMC showed significantly longer RT than the TD controls (*P* < 0.003) (Table 5).

**Table 5:** The duration of retrograde flow (RT) in the great saphenous vein (GSV) of lower limbs (*n* = 60 each) in children with myelomeningocele (MMC group) and typical developing children (TD group).

**Position**	**GSV segment**	**RT (s) MMC (*****n*** **=** **60)**			**RT (s) TD (*****n*** **=** **60)**			**Statistical tests *P*-values**
		**Mean (sd)**	**Median**	**Range**	**Mean (SD)**	**Median**	**Range**	
HP	P1	0.22 (0.08)	0.20	0.00–0.70	0.04 (0.04)	0.00	0.00–0.12	*U* = 1.29; *P* = 0.003
	P2	0.18 (0.08)	0.16	0.00–0.50	0.06 (0.04)	0.00	0.00–0.12	*U* = 1.29; *P* = 0.003
	P3	0.27 (0.06)	0.25	0.00–0.50	0.09 (0.04)	0.00	0.00–0.13	*U* = 3.52; *P* < 0.001
	P4	0.24 (0.11)	0.22	0.00–0.50	0.06 (0.05)	0.00	0.00–0.12	*U* = 1.12; *P* < 0.001
VP	P1	0.29 (0.11)	0.27	0.00–0.50	0.04 (0.05)	0.03	0.00–0.10	*U* = 3.60; *P* < 0.001
	P2	0.27 (0.21)	0.25	0.00–0.50	0.04 (0.04)	0.00	0.00–0.10	*U* = 3.52; *P* < 0.001
	P3	0.46 (0.16)	0.44	0.00–1.00	0.08 (0.04)	0.00	0.00–0.30	*U* = 3.60; *P* < 0.001
	P4	0.36 (0.16)	0.32	0.00–1.50	0.06 (0.04)	0.00	0.00–0.20	*U* = 3.60; *P* < 0.001

*HP, Horizontal position; VP, vertical position; GSV, great saphenous vein; P1, proximal thigh level; P2, medial thigh level; P3, upper leg level; P4, lower leg level. U = Mann–Whitney test for independent samples*.

*Means (SD), median, and range of the outcome (95% CI) and comparison between the groups (statistical tests and P-values)*.

In contrast to that in controls, the RT in children with MMC was significantly different between studied positions (HP vs. VP) (Table 6). After the MMC patient's transition from HP to VP, the RT in all four segments significantly increased (Table 6).

**Table 6:** The duration of retrograde flow (RT) in the great saphenous vein (GSV) of lower limbs (*n* = 60 each) in children with myelomeningocele (MMC group) and typical developing children (TD group) in the horizontal position and vertical position.

**Group**	**GSV segment**	**RT (s) Horizontal position**			**RT (s) Vertical position**			**Statistical tests *P*-values**
		**Mean (sd)**	**Median**	**Range**	**Mean (SD)**	**Median**	**Range**	
MMC	P1	0.22 (0.08)	0.20	0.00–0.70	0.29 (0.11)	0.27	0.00–0.50	*U* = 3.25; *P* = 0.004
	P2	0.18 (0.08)	0.16	0.00–0.50	0.27 (0.21)	0.25	0.00–0.50	*U* = 3.76; *P* = 0.001
	P3	0.27 (0.06)	0.25	0.00–0.50	0.46 (0.16)	0.44	0.00–1.00	*U* = 2.81; *P* = 0.005
	P4	0.24 (0.11)	0.22	0.00–0.50	0.36 (0.16)	0.32	0.00–1.50	*U* = 2.92; *P* = 0.001
TD	P1	0.04 (0.04)	0.00	0.00–0.12	0.05 (0.05)	0.03	0.00–0.10	*U* = 1.31; *P* = 0.565
	P2	0.06 (0.04)	0.00	0.00–0.12	0.06 (0.04)	0.00	0.00–0.10	*U* = 1.29; *P* = 0.589
	P3	0.06 (0.04)	0.00	0.00–0.13	0.09 (0.04)	0.00	0.00–0.30	*U* = 1.27; *P* = 0.608
	P4	0.08 (0.05)	0.00	0.00–0.12	0.11 (0.04)	0.00	0.00–0.20	*U* = 1.52; *P* = 0.543

*GSV, Great saphenous vein; P1, proximal thigh level; P2, medial thigh level; P3, upper leg level; P4, lower leg level. U = Mann–Whitney test for independent samples*.

*Means (SD), median, and range of outcome (95% CI) and comparison between study positions*.

**Figure 1:**
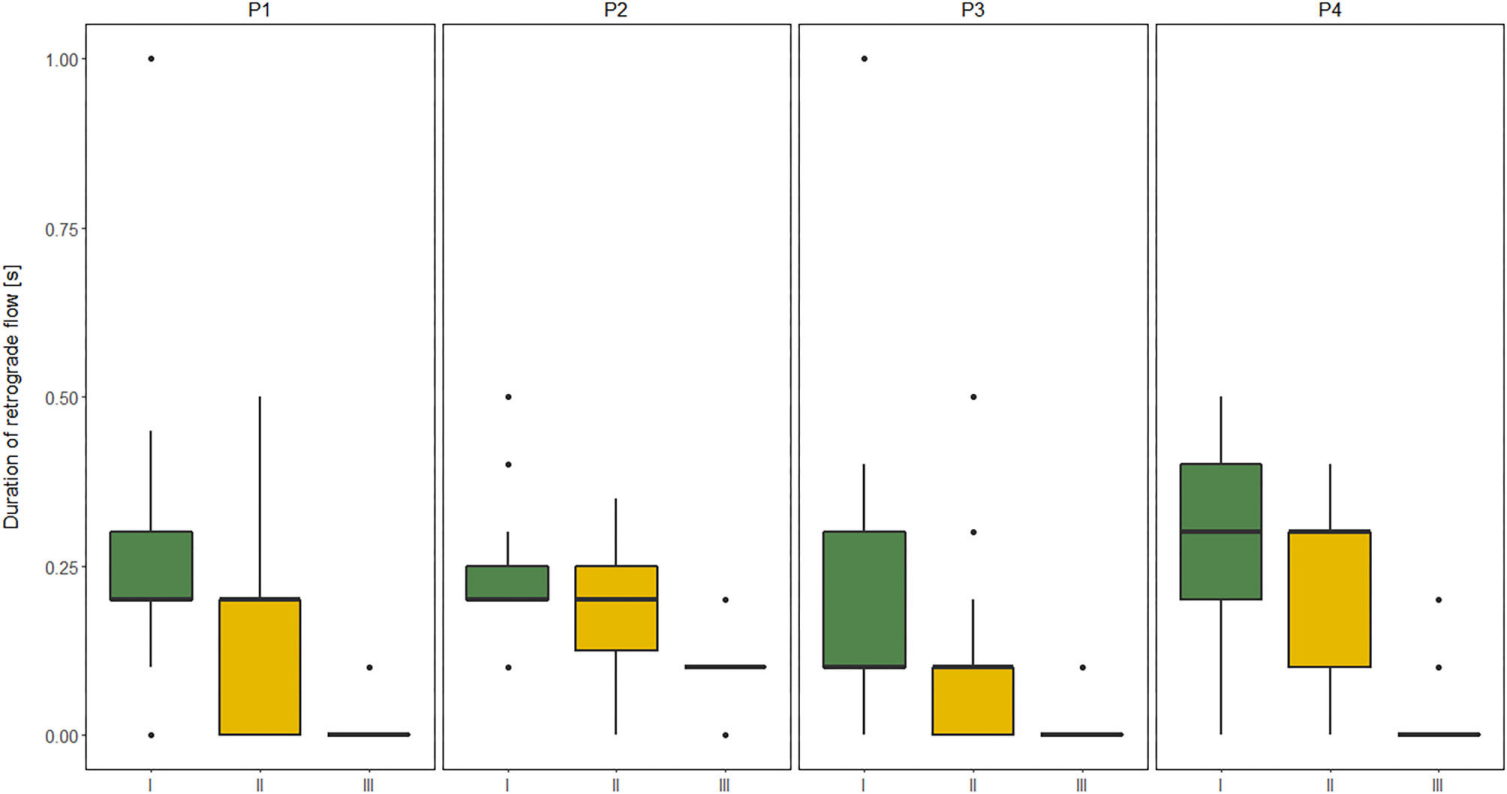
Comparisons between subgroup: I, II, and III level of Gross Motor Function Classification System (GMFCS) in relation to the duration of retrograde flow (RT) evaluated at four sites of Great saphenous vein (GSV): proximal thigh level (P1); medial thigh level (P2); upper leg level (P3); lower leg level (P4) of lower limbs (*n* = 60 each) measured in vertical position in children with myelomeningocele (MMC group). Kruskal–Wallis test for independent samples.

**Figure 2:**
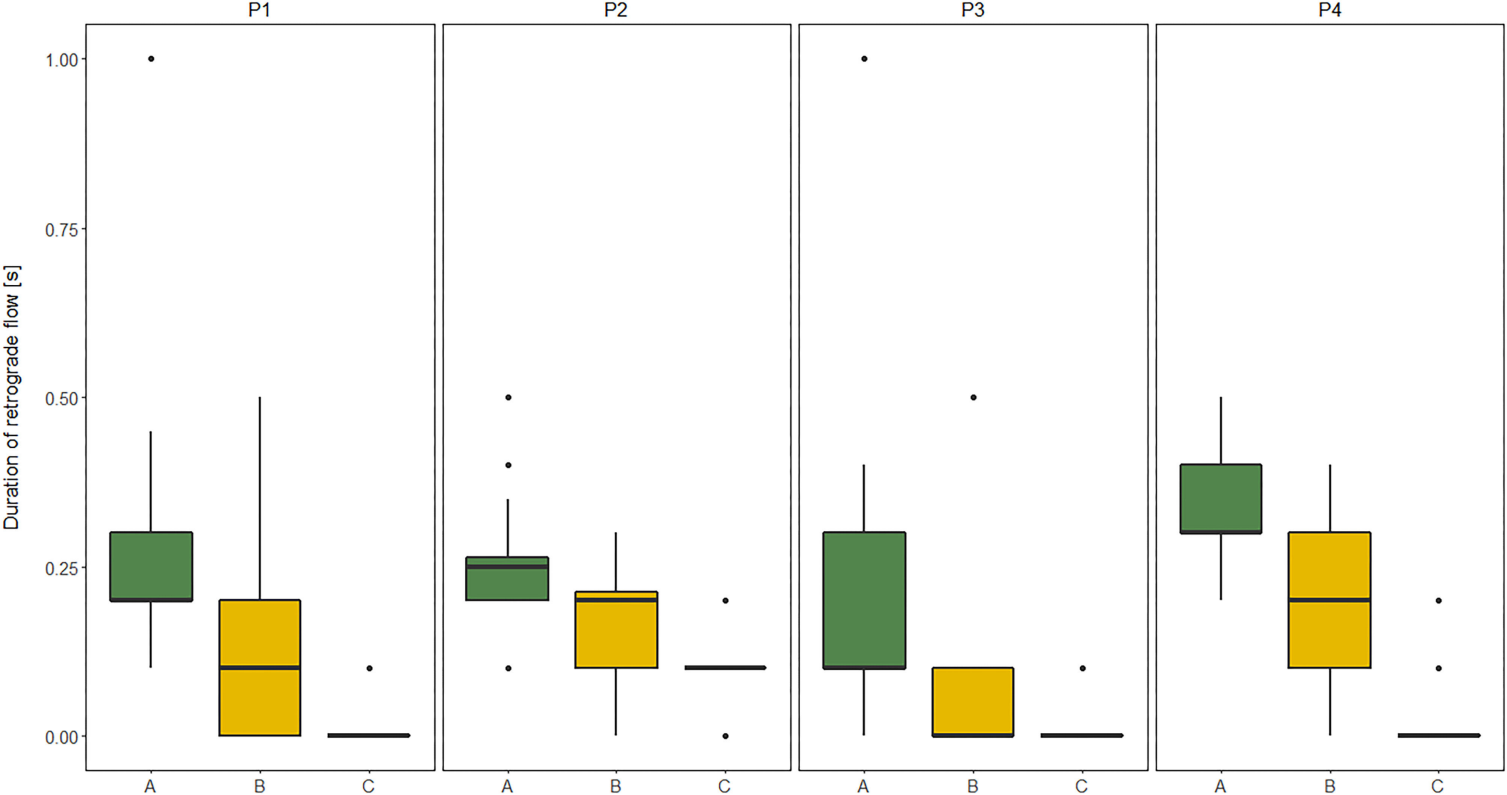
Comparisons between subgroup: A (high lumbar), B (low lumbar), and C (sacral) level of the spinal cord defect in relation to the duration of retrograde flow (RT) evaluated at four sites of Great saphenous vein (GSV): proximal thigh level (P1); medial thigh level (P2); upper leg level (P3); lower leg level (P4) of lower limbs (*n* = 60 each) measured in vertical position in children with myelomeningocele (MMC group). Kruskal–Wallis test for independent samples.

The duration of RT in GSV and its relation to clinical severity of MMC, expressed by the level of the spinal cord defect (subgroups A, B, C), and their relation to functional limitation, expressed by the level of GMFCS (I, II, III) are shown in Figures 1, 2.

The GSV reflux (RT of at least 0.5 s) was recognized exclusively in the MMC group: 59 of 240 (25%) cases during examination in HP and 72 of 240 (30%) in VP. The reflux was more common in the peripheral segments (P3, P4) than the proximal segments (P1, P2) of GSV [respectively: 37 [15% cases] and 22 [9% cases] of 240 in HP and 45 [19% cases] and 27 [11% cases] of 240 in VP, both *P* < 0.05]. Moreover, data analysis showed that the prevalence of GSV reflux (in VP) was significantly higher in subgroups A (51 of 96 cases, 53%) and B (18 of 80, 23%) than subgroup C (3 of 64, 4%) (χ^2^ = 42.7, *P* < 0.0001; and χ^2^ = 63.4, *P* < 0.0001, respectively). The duration of RT in GSV and their association with gross motor function score (GMFM) and strength of particular muscle groups of lower limbs (MMT score) in the MMC group are shown in Table 7.

**Table 7:** Spearman's correlation coefficients between the duration of retrograde flow (RT) in the great saphenous vein (GSV) and functional independence score (GMFM) and muscle strength (MMT) of lower limbs in children with myelomeningocele.

**GSV segment**	**Measurement position**	**GMFM**	**HF**	**KE**	**ADF**	**LTE**	**APF**
		**R coefficient/*****P*****-value**					
P1	HP	−0.56^**^	–0.42^*^	–0.62^**^	–0.67^**^	–0.69^**^	–0.66^**^
	VP	–0.61^**^	–0.54^*^	–0.62^**^	–0.75^**^	–0.73^**^	–0.76^**^
P2	HP	–0.58^**^	–0.49^*^	–0.64^*^	–0.65^**^	−0.63^**^	–0.43^*^
	VP	–0.56^**^	–0.52^*^	–0.63^*^	–0.71^**^	–0.69^**^	–0.38^*^
P3	HP	–0.66^**^	–0.50^*^	–0.50^*^	–0.72^**^	–0.74^**^	–0.69^**^
	VP	–0.61^**^	–0.50^*^	–0.52^*^	–0.69^**^	–0.62^*^	–0.66^**^
P4	HP	–0.71^**^	–0.49^*^	–0.54^*^	–0.65^**^	−0.63^**^	–0.43^*^
	VP	–0.76^**^	–0.52^*^	–0.53^*^	–0.71^**^	–0.69^**^	–0.38^*^

* *P < 0.01*;

** *P < 0.05*;

*HP, Horizontal position; VP, vertical position; P1, proximal thigh level; P2, medial thigh level; P3, upper leg level; P4, lower leg level; HF, muscle strength of hip flexors; KE, knee extensors; ADF, ankle dorsiflexors, LTE, long toe extensors, APF, ankle plantar flexors*.

## Discussion

The present study described the differences in GSV properties between children with MMC children and their TD peers. Our main finding was recognition of the discrepancy between duration of RT of the GSV in children with MMC and TD peers. The results showed that the duration of RF time in GSV was significantly longer in the MMC group than the TD group. Our data analysis revealed that children with MMC presented three-times and five-times higher durations of RT in the proximal GSV segments (P1, P2) and distal GSV segments (P3, P4), respectively, in both measurement conditions (HP and VP) than the TD group (Table 5).

Moreover, our results showed a few significant relationships between the GSV insufficiency and neurological impairment, deficit of lower limb muscle strength, and GMFM limitation of patients with MMC. Our study recognized that the duration of RT of GSV was negatively associated with lower limb muscle strength. Good and mostly moderate correlations were observed between the RT of all four GSV segments and the strength of leg muscle groups (ankle dorsiflexors and long toe extensors) and the thigh muscle groups (hip flexors and knee extensors) of lower limbs, respectively (Table 7).

The similar dependencies were concerned the RT of all four GSV segments and functional limitations in MMC children. The RT of proximal segments of GSV (P1, P2) showed good correlation to GMFM, while the RT of distal segments of GSV (P3, P4) was only moderately correlated with GMFM (Table 7). Furthermore, patients classified as GMFCS level III showed significantly shorter RF duration in all four levels of GSV in both HP and VP than other groups (GMFCS levels I and II) (Figure 1). Third, the RF time of GSV was significantly longer in children classified to have a high lumbar level (subgroup A) than in those classified to have low lumbar (subgroup B) and sacral levels (Figure 2).

Moreover, above 45% of children with MMC showed evidence of antegrade flow followed by RT (after a quick, firm compression of a peripheral segment of the GSV) in at least one segment of GSV. Because there are no reference standards for vein reflux characteristics for the pediatric population, results are usually extrapolated from studies conducted on adult subjects. Although, in the present study, the GSV reflux was documented when a significant amount of RT was found, the duration of at least 0.5 s RT was recognized in over 18% of all duplex scanning images for GSV in the MMC group. None of the symptoms of venous insufficiency were observed in the TD group.

Most cases of GSV reflux in MMC were detected in the above-knee segments, mainly in the lower leg level (P4) and rarely in the upper leg level (P3) and thigh segments of GSV (P1 or P2). Combined reflux including the upper and lower leg levels (P4 and P3) were found in half of the leg reflux cases. The reflux occurred in the thigh and leg segments of GSV in only very few cases. Reflux in the GSV, most of all, was prevalent among participants who presented with high lumbar level (>50% of all cases).

Interestingly, the GSV reflux in MMC children was significantly frequent during examination in VP vs. HP. Additionally, after transition from HP to VP, the duration of RT of all four GSV segments showed significant increase (Table 6).

To our best knowledge, no other studies have examined the venous properties of the leg in MMC, which makes it difficult to compare our results with previously published literature.

To conclude, our data suggest the following:

1. Children with MMC showed increased duration of RT of both the proximal and peripheral sites of GSV, as compared with the TD peers.
2. In many children with MMC, reflux (RT of at least 0.5 s) occurred in at least one segment of GSV.
3. The prevalence of GSV reflux in peripheral segments was significantly higher than in the proximal segments.
4. The severity of MMC (expressed by higher level of the spinal cord defect), deficit of thigh and leg muscle strength, and lower functional independence negatively influenced the GSV sufficiency in patients with MMC.
5. Gravity directly influenced GSV reflux occurrence and reflux hemodynamic parameters in MMC.

Although it is known that individuals with SB are prone to secondary complications such as decubitus ulcers and poor wound healing, the venous properties in individuals with MMC have not been studied. The present study confirmed that venous insufficiency is common in children with MMC. These data highlighted the tendency to develop reflux in the superficial veins such as in the below-knee segment of the GSV. Although the observed positional differences (between HP and VP) of RT duration of GSV in children with MMC suggest that gravitational forces play a significant role, this is not enough to explanation for reflux flow in GSV. The obtained results showed that the prevalence and severity of venous insufficiency were clearly associated with the clinical severity of MMC.

While valvular insufficiency is the principal cause for the development of primary venous reflux in adults, the etiologic mechanism of venous insufficiency in children with MMC is not adequately understood. It is likely that poor venous properties in these children may be attributed to disturbances in the development of biological adaptations such as muscle pumps and venous valves, with the evolution of supportive fascial structures. A few recent studies clearly suggest that one of the factors to contribute to edemas, trophic disorders, and decubities in individuals with MMC can be disturbances blood flow in the denervated muscles of lower limbs (6, 7).

Signs of venous insufficiency developing in children with MMC may be an indicator of subsequent development of more severe venous disease. Signs such as increased time of RT of both proximal and peripheral sites of GSV likely suggest that these patients should take precautions to minimize further development of venous disease (i.e., compression using garments or bandaging, or Kinesio taping).

Given that the Doppler ultrasound tests not have been validated among the pediatric population, the best solution may be to provide an absolute value of venous characteristics. Although time of RT is a good parameter to identify the presence of reflux, it is not useful to quantify the same. Nevertheless, our preliminary findings encourage to further research of venous reflux in children with MMC with larger sample sizes and extended protocols of other hemodynamic characteristic of GSV (i.e., reflux volume and reflux volume flow rate, diameter of the vein and its cross-sectional area).

## Conclusion

These findings may help guide further prospective studies to more clearly delineate better understand aspects concerning the risk of developing venous insufficiency in children with MMC and determine better screening, prevention, and treatment algorithms for venous insufficiency in patients with SB.

## Study Limitations

Given that, to our best knowledge, this is the first study to examine the venous properties of the leg in children with MMC, few limitations should be acknowledged when interpreting the results. First, there are no established standards yet for ultrasound-Doppler examination of the venous properties of the leg in the pediatric population. In this study, we extrapolated and partially modified the standards of GSV examination from studies performed in adults. Second, there is a lack of normative values of hemodynamic characteristics for the pediatric population.

Additionally, there is no there is no consensus in the literature about the normative values of hemodynamic characteristics of GSV, such as duration of RT, reflux time, reflux volume, or reflux volume flow rate. Depending on its extent, the reflux was classified as proximal if confined to the veins above the knees; as distal, if confined to the GS veins below the knees; or both proximal and distal. According to the recommendations of the Polish Society for Vascular Surgery and Polish Society of Phlebology (based on the normal values proposed by Labropoulos) (15), reflux, in this study, was documented when a significant amount of antegrade flow was found. However, the criterion of 0.5-s RT was used to identify pathological venous reflux (16, 17). Last, the small sample size of patients with MMC (n = 30) limits the power to establish stronger associations. This should be addressed in future research by using a multicenter, prospective study design with a larger sample size.

## Data Availability Statement

The original contributions presented in the study are included in the article/supplementary material, further inquiries can be directed to the corresponding author/s.

## Ethics Statement

The studies involving human participants were reviewed and approved by The Ethical Committee of the Medical University of Silesia (KNW/0022/KB1/37/18). Written informed consent to participate in this study was provided by the participants' legal guardian/next of kin.

## Author Contributions

MD-S and ASz contributed to the conception and design of the study. MD-S, ASz, and DZ contributed to the acquisition and analysis of data. MD-S, ASz, MP, LS, ASi, and MH-D contributed to drafting and editing the manuscript text. All authors agreed on the final version of the manuscript. All authors contributed to the article and approved the submitted version.

## Conflict of Interest

The authors declare that the research was conducted in the absence of any commercial or financial relationships that could be construed as a potential conflict of interest.
